# The role of genetic distance in modulating intraspecific plant–soil interactions among a diverse assemblage of tropical tree species seedlings

**DOI:** 10.1098/rspb.2025.3199

**Published:** 2026-10-21

**Authors:** Rosa I. Soria Penafiel, Karla Zambrano, Carlos Aulestia, Gonzalo Rivas-Torres, Jordan Karubian, Sunshine A. Van Bael

**Affiliations:** ^1^ Ecology and Evolutionary Biology, Tulane University, New Orleans, LA, USA; ^2^ Facultad de Ciencias Exactas, Naturales y Ambientales, Pontificia Universidad Católica del Ecuador, Quito, Pichincha, Ecuador; ^3^ Fundación para la Conservación de los Andes Tropicales (FCAT), Quinindé, Esmeraldas, Ecuador; ^4^ Estación de Biodiversidad Tiputini, Colegio de Ciencias Biológicas y Ambientales, Universidad San Francisco de Quito, Quito, Pichincha, Ecuador

**Keywords:** conspecific negative density dependence, double-digest restriction-site-associated DNA sequencing, genetic variation, intraspecific density dependence, Janzen–Connell hypothesis, plant growth, pathogenic and mutualistic soil microbes

## Abstract

Microbial specialization on host species can promote plant diversity through plant–soil interactions that affect seedling survival and performance. While these processes are typically studied at the interspecific level, emerging evidence suggests that plant–soil interactions can be genotype-specific, contributing to intraspecific density dependence (iDD). However, iDD remains poorly understood in tropical tree species, particularly with respect to its strength and frequency. We hypothesized that genetic distance among conspecific trees should modulate the strength of iDD, and the direction of the effect—positive or negative seedling response—should reflect the net balance of plant–microbe antagonistic and mutualistic interactions. Using a reciprocal planting experiment across eight tropical tree species, we show that genetic distance between conspecific individuals significantly influences seedling responses, consistent with iDD. Yet, the direction of this effect was variable: *Castilla tunu* showed higher survival and *Wettinia aequalis* greater biomass in soils from more genetically distant conspecifics, consistent with negative iDD; *Inga marginata* exhibited positive iDD; five species exhibited no pattern. These findings demonstrate that genotype-specific plant–soil interactions can generate biologically relevant but variable impacts on seedling responses. This highlights the need to further investigate the drivers and ecological consequences of intraspecific genetic variation in plant–soil interactions and iDD in tropical forests.

## 1. Introduction

Understanding the mechanisms that maintain species diversity and relative abundance is a central aim in ecology and evolutionary biology. One key stabilizing mechanism in tropical forests and other habitats is thought to be conspecific negative density dependence (CNDD), which occurs when fitness declines with increasing densities of conspecific adults [1–3]. The Janzen–Connell hypothesis [4,5] provides a biological explanation for CNDD, proposing that host-specific enemies (e.g. soil microbes or herbivores) accumulate near reproductive adults, reducing seedling survival in areas of high conspecific density. This process decreases spatial aggregation of conspecifics and facilitates the establishment of other species, promoting diversity and species coexistence [2,6,7]. Although these dynamics are typically considered at the species level, increasing evidence suggests that soil microbes may also exhibit specialization among host genotypes within host species [8–11]. However, the extent to which such microbial specialization within species contributes to density-dependent demographic patterns among conspecific individuals in tropical forests remains poorly understood.

Among plants, soil microbes are thought to play a particularly important role in shaping patterns of CNDD [6,12,13] and these dynamics in turn form part of broader plant–soil interactions, in which plants influence soil microbial communities in ways that reciprocally affect their own growth and reproduction, as well as that of conspecifics and heterospecifics [14]. In tropical forests, soil-borne fungal pathogens are recognized as key agents of CNDD, contributing to species coexistence through negative plant–soil interactions [12,15,16]. While much research has focused on the stabilizing role of pathogens via negative density dependence among conspecific plants [9,10,13,17,18], recent studies suggest that microbes can also destabilize plant coexistence when associations with mutualistic soil microbes enhance host performance (i.e. self-promotion) [7,12,19,20], leading to positive density dependence and competitive exclusion [21,22]. However, how commonly plant–soil interactions generate negative versus positive density-dependent effects among plants within tropical tree species remains uncertain [21].

Emerging evidence from both crops [23,24] and tropical forest species [8–11] suggests that microbial specialization can occur not only between species but also within species, with both pathogens and mutualists exhibiting variation in the strength of plant–soil interactions among conspecific genotypes. Despite growing recognition of this phenomenon, the mechanisms underlying these interactions and their role in shaping intraspecific density dependence patterns in tropical forests remain poorly understood. In this context, we use the term *intraspecific density dependence* (iDD) to refer to changes in demographic performance that arise from genotype-specific plant–soil interactions among conspecific individuals [7,25,26]. Thus, iDD can be viewed as a finer-scale analogue of CNDD operating among individual conspecific plants that differ genetically. However, while CNDD generally reflects negative density-dependent effects associated with the accumulation of host-specific pathogens, iDD may generate either *negative* or *positive* demographic responses depending on the net balance of antagonistic versus mutualistic genotype-specific plant–soil interactions.

Previously, researchers have studied within-species microbial specialization in wild species by comparing seedling performance in maternal versus non-maternal conspecific soil or close versus distant soil [11,18,25]. For example, Eck *et al.* [10] in a shade house experiment in Panama, found that seedlings of the native tree species *Virola surinamensis* had reduced performance in the soil microbial community of their maternal tree compared to the soil microbial community of a non-maternal tree from the same population, suggesting that highly specialized pathogens were the mechanism reducing seedling performance in maternal soils. This study captured the net effects of antagonists versus mutualists on iDD (i.e. a negative effect) without disentangling their distinct or synergistic contributions [21].

Recent studies have included metrics of genetic distance among conspecifics to evaluate how genetic variation might influence the strength and direction of intraspecific plant–soil interactions. A study from Ecuador focusing on the palm *Oenocarpus bataua* found that older seedlings (>2 yr) had lower survival when they were more genetically similar to nearby conspecific adults, suggesting a rare-genotype survival advantage potentially mediated by interactions with soil microbes. Interestingly, this relationship varied with seedling age: younger seedlings (<2 yr) tended to survive better when in proximity to a conspecific [26]. Similarly, a study focused on *Beilschmiedia roxburghiana* in a tropical rain forest in China revealed that individuals surrounded by closely related conspecific neighbours exhibited lower relative growth rates, suggesting that seedling survival and performance are modulated by genetic distance among neighbouring conspecifics potentially through competition or shared natural enemies [27]. Liu *et al.* [17] reported that when seedlings of *Castanopsis fissa* and *Canarium album* were treated with soil biota from genetically distant conspecific trees, the negative effect of the soil biota on seedling survival decreased with increasing genetic dissimilarity. Building from this body of recent work, we set out to determine whether the degree to which genetic distance among conspecifics can help explain the magnitude and direction of genotype-specific plant–soil interactions in wild species, and how widespread this phenomenon might be among tropical forest trees.

Given this evidence that plant–soil interactions can be mediated through individual-level, genotype-specific variation to generate iDD patterns, we hypothesize that seedling survival and performance are modulated by genetic distance among conspecific hosts. Genetic similarity between seedlings and more closely related conspecific adults may increase the likelihood of encountering shared genotype-specific antagonists, potentially resulting in negative plant–soil interactions. Conversely, if interactions are driven by genotype-specific mutualists, genetically similar conspecifics may have a higher likelihood of associating with similar mutualists, leading to positive plant–soil interactions. Consequently, populations dominated by genotype-specific antagonistic soil communities may exhibit stronger negative iDD, whereas populations where beneficial mutualists play a larger role may exhibit positive or weaker density-dependent responses. Thus, the direction of these responses (positive versus negative) may reflect the net outcome of potentially opposing effects of antagonistic versus mutualistic genotype-specific plant–soil interactions. These dynamics may influence iDD in distinct ways. More specifically, the net impact of genotype-specific plant–soil interactions on a given plant species could be positive, or negative, or could effectively balance each other out such that no net impact is detectable (figure 1). The strength and direction of iDD may also vary among species and among individuals within species. Such variation could arise from differences in levels of genetic diversity, species-specific traits influencing plant–soil interactions [9,24] or variation in the degree of genotype specificity within associated soil microbial communities.

**Figure 1.**
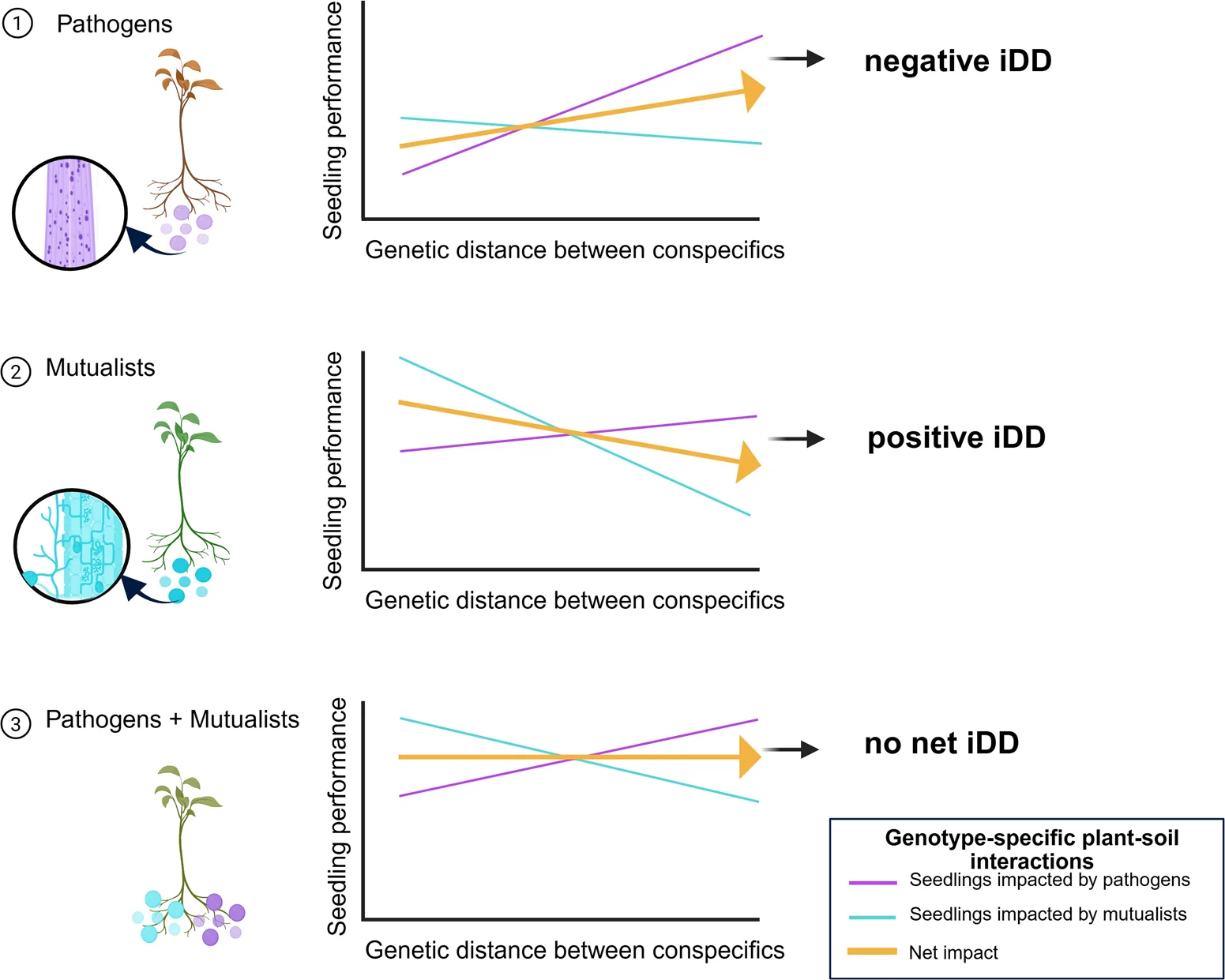
Conceptual model illustrating how genotype-specific plant–soil interactions among conspecifics can generate different intraspecific density dependence patterns: (1) negative intraspecific density dependence (negative iDD), if seedling performance in conspecific soil increases with genetic distance (pairwise genetic distance between the maternal seed tree and the conspecific soil-source tree) from conspecific adults, this suggests that closely related individuals experience stronger negative plant–soil interactions, consistent with genotype-specific antagonists that outweigh potential mutualistic benefits; (2) positive intraspecific density dependence (positive iDD), if seedling performance in conspecific soil decreases with genetic distance, indicating that seedlings benefit from associating with genetically similar individuals, consistent with mutualistic effects that outweigh antagonistic interactions; (3) neutral interactions (no net iDD), if seedling performance is unaffected by genetic distance, it may also indicate that antagonistic and mutualistic interactions co-occur and counterbalance each other at the individual level, resulting in a net neutral effect.

Documenting the strength, frequency and underlying drivers of iDD has the potential to advance our ability to understand the factors that shape tropical tree distributions and community structure and, by extension, emergent patterns of taxonomic and genetic diversity. To address this knowledge gap, we conducted a fully crossed shade house experiment on seedling survival involving a taxonomically and ecologically diverse assemblage of eight tree species from a tropical forest in northwest Ecuador. Our overarching goal was to assess how genetic distance between conspecifics influences genotype-specific plant–soil interactions and iDD. Our specific objectives were to: (i) assess the degree to which the strength of iDD, as measured using seedling survival and performance, covaries with genetic distance between maternal seed sources and (ii) determine the direction and magnitude of iDD across species, which may reflect the net effects of antagonistic versus mutualistic genotype-specific plant–soil interactions.

## 2. Material and methods

### (a) Study site and focal species

This research was conducted in the Chocó biogeographic zone of northwestern Ecuador, a global biodiversity hotspot facing severe habitat loss and declining species abundance owing to deforestation and other anthropogenic pressures [28]. Fieldwork and experimental setup took place at the Fundación para la Conservación de los Andes Tropicales (FCAT; 00°23′28′′ N, 79°41′05′′ W), a 734-hectare private reserve located in Esmeraldas Province. The region experiences average temperatures between 23 and 25°C and annual precipitation of approximately 2500–3000 mm, with a wet season spanning from late December to June [29].

We selected eight tree species from diverse tropical families that are currently part of an ongoing forest restoration programme at FCAT: *Inga marginata* and *Inga edulis* (Fabaceae); *Castilla tunu* and *Castilla elastica* (Moraceae); *Wettinia aequalis*, *Bactris setulosa* and *Socratea exorrhiza* (Arecaceae); and *Vismia sprucei* (Hypericaceae). Seeds were collected for all eight species. The selected species produce small to medium-sized seeds (approx. 7–20 mm) that are animal-dispersed [30]. For members of the Arecaceae family, seed production peaks from July to November [31], whereas the remaining species can fruit multiple times annually [32].

### (b) Seeds and live soil inoculum collection

Between five and eight adult trees per species were selected across a range of spatial scales from a few metres to several kilometres apart, with pairwise distances determined by availability of concurrently fruiting adults of a given species during the project period. The geographic coordinates of each tree were recorded using a handheld GPS (figure 2). Approximately 100 seeds were collected from each tree on a day between July 2021 and March 2022 (depending on species) and transported to the FCAT research station, where they were soaked in water and the epicarp and mesocarp were manually removed with sterilized pliers. The cleaned seeds were surface-sterilized in 10% bleach for 5 min, followed by a 5-min rinse in 20% ethanol [33]. Tools were sterilized with 70% ethanol between species. Seeds were germinated directly into bags with autoclaved bulk soil (see below).

**Figure 2.**
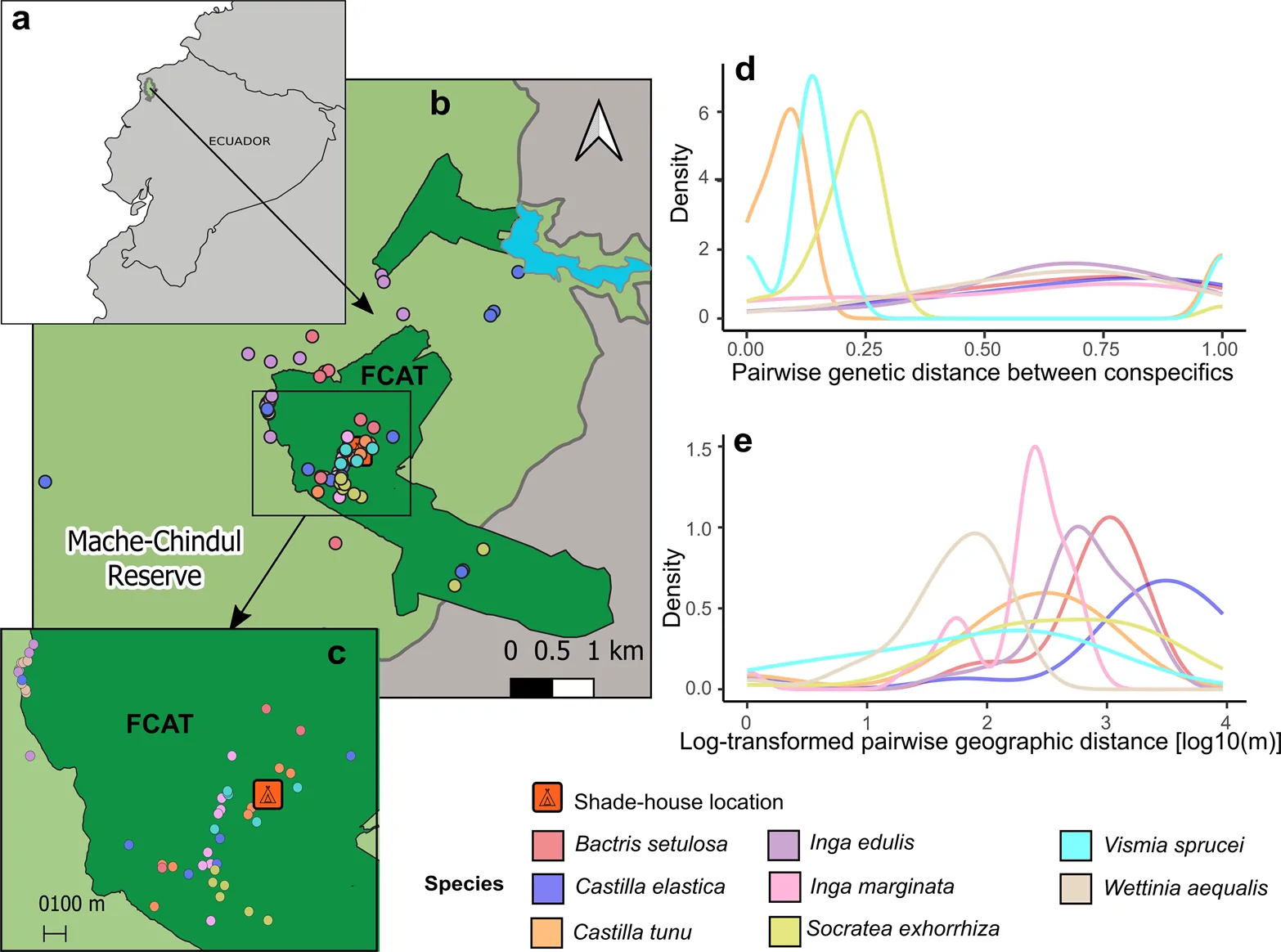
Overview of the study area and the distribution of the focal species. Inset (a) shows the location of study area in and around the FCAT reserve, near the western coast of Ecuador. (b) The location of maternal trees from which seeds and soil inoculum were collected. (c) Inset zoom of the FCAT reserve highlighting areas with closely spaced maternal trees, providing a clearer view of their spatial arrangement. (d) Kernel density estimates of pairwise normalized genetic distances among conspecific adults, where values near 0 indicate high genetic similarity and values near 1 reflect greater genetic divergence. (e) Kernel density estimates of pairwise log-transformed geographic distances among conspecific adults, illustrating patterns of spatial dispersion within species.

Composite live soil inoculum samples were obtained from each adult tree by collecting three subsamples within a 2 m radius from the trunk at 5 cm depth. These subsamples were homogenized and stored in Ziploc bags for a maximum of 24 h before use. All tools used for soil collection were sterilized between samples with soap followed by 70% ethanol. Although inoculum was collected from standardized depths and distances, we acknowledge that microbial community composition may vary owing to unmeasured environmental factors such as tree age, microhabitat or recent rainfall. These factors were not controlled but are partially accounted for by including inoculum source as a random effect in statistical models.

#### (i) Bulk soil preparation

Bulk soil for the experiment was sourced from topsoil (top 20 cm layer) from older secondary forest near the FCAT station. Soil was sieved through a 5 mm mesh to remove debris, then bagged and transported (approx. 300 kg) to INIAP (Instituto Nacional de Investigaciones Agropecuarias, Ecuador) for sterilization by autoclaving. Sterilized soil was returned to the FCAT reserve in bags for experimental use.

#### (ii) Fully crossed planting experiment

For each species, a fully crossed planting design was conducted. Seeds from each maternal tree were planted into pots containing one of three inoculum types: (1) maternal soil inoculum collected from the same maternal tree, (2) soil inoculum collected from other conspecific adult trees or (3) soil without inoculum which was used as a control. Seedling pots consisted of 1 l low-density polyethylene bags (0.09 mm thick), each filled with autoclaved soil. One seed per pot was sown at a depth of 2 cm. Plants were monitored daily and watered twice per week. When 75% of the plants of a given species had germinated, the adult trees were visited to collect the inoculum (see above). A supplement of 50 ml of the designated live soil inoculum was added to the top layer of each pot. No additional soil was added to control seedlings.

Each seed per soil inoculum combination was replicated eight times, totalling 3568 seedlings as detailed in supplementary material, table S1a. Seedling pots were arranged in a randomized block design. For each species, eight raised beds served as blocks, and each block contained one replicate of every seed × soil inoculum combination for that species. Raised beds were located under a shade house constructed from 0.2 mm LDPE sheeting and dual-layer high-density polyethylene netting (approx. 80% shade).

#### (iii) Plant growth

Seedlings were harvested approximately 6 months post-germination. Each was measured for stem height and separated into above- and below-ground biomass by cutting approximately 2 mm above the soil line. Roots were gently washed with water using a hose over nested 2 and 0.5 mm sieves to remove adhering soil while minimizing root loss. Cleaned roots were recovered with forceps, then carefully transferred to labelled paper bags. Above- and below-ground samples were then oven-dried at 50°C for 4 days. Dry above-ground biomass was measured to the nearest 0.01 g using a precision scale (Ohaus Scout, Parsippany, New Jersey), while dry below-ground biomass was measured to the nearest 0.0001 g with a precision scale (Ohaus Analytical Plus, Parsippany, New Jersey).

### (c) Estimation of genetic distances

Genetic distances among conspecific adult trees were estimated using double-digest restriction-site-associated DNA sequencing (ddRADseq) [34] and processed through the STACKS v2.2 pipeline [35,36]. Leaf tissue was collected from each adult tree in May 2022 and dried in paper envelopes with silica gel. In November 2022, dried samples were transferred from FCAT station to Tulane University (New Orleans, USA), where DNA was extracted using a DNeasy PowerSoil Pro Kit (Qiagen, Hilden, Germany), following the manufacturer's protocol [37]. DNA extracts were sent to the Tulane National Primate Research Centre (TNPRC), where libraries were constructed using a modified ddRADseq protocol [38]. In short, genomic DNA was digested with EcoRI and MspI (New England Biolabs, Ipswich, MA, USA), and adapters were ligated to digested fragments. Size selection (300–600 bp) was performed using AMPureXP magnetic beads (Beckman Coulter, Indianapolis, IN, USA). Libraries were amplified, purified and sequenced using paired-end 300 bp reads on an Illumina NextSeq 2000 platform (600-cycle kit).

Raw reads were demultiplexed and processed with STACKS v2.2 to identify single nucleotide polymorphisms (SNPs) de novo for each species. Parameter optimization for ustacks (-m, -M) and cstacks (-n) was performed with a subset of individuals per species [39], comparing outputs based on the number of variant sites recovered, genetic diversity metrics and patterns of genetic distance in multidimensional scaling plots (supplementary material, table S1a). For analyses including all samples per species, all individuals within each species were treated as a single population and diversity metrics were obtained (polymorphic sites, heterozygosity and nucleotide diversity -*π*-). Loci were retained if present in ≥80% of individuals (-r 0.8), with a minimum minor allele frequency of 0.05 (–min_maf), and only one SNP per locus was retained to reduce linkage (–write-random-snp) [38]. Pairwise genetic distances between conspecific adults were calculated as Euclidean distances between individuals in principal component analysis (PCA) space, using the first three principal components generated in PLINK from SNP data exported by the populations module of STACKS. This PCA-based distance was used as a reduced-dimensional measure of genetic dissimilarity among conspecific adults, following previous use and evaluation of multi-axis PCA-based genetic distance metrics and PCA-derived Euclidean genetic distances in genetic-structure and landscape-genetic analyses [40,41]. This was implemented in R v4.4.1 [42] using the dist() function from the stats package. PCA ordinations based on the first two principal components are shown in figure S1a–e, supplementary material. For species with insufficient sequencing depth (<3×; *V. sprucei*, *S. exorrhiza* and *C. tunu*), genetic distances were estimated using *k*-mer based distances with Mash [43], using parameters -m 3 and -s 1000. Details of the genetic distance estimation methods applied across species are summarized in table S1b, supplementary material. In this study, the term *genetic distance* refers to a quantitative measure of genomic dissimilarity between conspecific adult trees, calculated from multilocus allele frequency data or *k*-mer profiles. It represents the degree of genetic divergence between the maternal tree from which a seed was collected and the conspecific adult tree from which soil inoculum was sourced. To ensure consistent interpretation in analyses that combined multiple species, genetic distances were normalized within each species by dividing by the maximum observed intraspecific distance. This normalized metric was used only in models and figures that integrated data across species. For species-specific analyses, untransformed genetic distance estimates were used. The relationship between geographic and genetic distance among conspecific adults is described in the supplementary material (supplementary 3).

### (d) Statistical analyses

All statistical analyses in this study were conducted in R [42]. Dry above-ground and below-ground biomass were summed to estimate total biomass, which was used as the primary measure of plant performance in all subsequent analyses. Total biomass provides an integrated estimate of overall seedling growth.

To evaluate within-species plant–soil interactions, we generated a categorical variable, *Soil inoculum* (Soil_inoc), with two levels: *maternal* (seedling grown in soil containing live inoculum from its maternal tree) and *other_conspecific* (seedling grown in soil containing inoculum from a genetically distinct conspecific adult). In addition, we included *Genetic distance* (Genetic_distance) as a continuous predictor to account for fine-scale genetic variation among conspecific adults. This variable represented the pairwise genetic distance between the maternal seed source and the conspecific adult tree used as the soil inoculum source. *Species* was included as a fixed effect to account for potential differences among host species in their responses to plant–soil interactions. Together, these variables allowed us to test whether seedling responses differed between maternal and non-maternal conspecific soils, whether plant–soil interactions vary as a function of genetic variation among conspecific adults and whether these patterns differ among species.

Seedling survival was modelled using generalized linear mixed-effects models (glmer(), family = binomial) from the lme4 package [44]. A set of candidate models including different combinations of fixed effects was compared using Akaike's information criterion (AIC). The model with the lowest AIC value, indicating the best fit to the data, included *Soil inoculum*, *Species*, their interaction and *Genetic distance* as fixed effects, with maternal identity, inoculum source and experimental block as random effects: Survival ∼ Soil_inoc*Species + Genetic_distance + (1|MaternalID) + (1|InoculumID) + (1|Block). Plant performance, quantified as total biomass, was analysed using linear mixed-effects models (lmer()) following the same model structure, which was also identified as the best-fitting model based on AIC comparisons. Both models were evaluated using Type II Wald chi-square tests with the Anova function in the R package car [45].

Autoclaved control treatments were excluded from the analysis because sterilized control soils contained no live soil inoculum derived from a conspecific tree; therefore, no corresponding inoculum source could be assigned and pairwise genetic distance could not be calculated for these treatments. Including them would have introduced observations lacking values for a key predictor variable into analyses explicitly designed to test the effect of genetic distance between maternal and inoculum source trees in intraspecific plant–soil interactions. To verify that seedling responses were driven by soil inoculum, we conducted additional models including control soils but excluding genetic distance as a predictor. The models included *Soil inoculum* and *Species* using the same random effects and structure described above (supplementary material, S5).

Marginal effects of fixed terms were visualized using the effects package [46]. *Post hoc* comparisons were conducted to assess significant differences between soil inoculum sources (maternal versus other conspecific) within each species using the emmeans package [47] on the response scale (type = ‘response’). Statistical significance on these comparisons was assessed using Wald *z*-tests for the binomial survival model and two-sided *t*-tests with Satterthwaite-approximated degrees of freedom for the linear model of total biomass.

To explore finer-scale specialization of soil microorganisms, we ran separate models for each species using maternal identity (rather than the broader inoculum category) as a predictor. These models tested whether seedlings exhibited differential responses to inoculum from genetically distant conspecific adults. Total biomass and survival were used as response variables in linear (lmer()) and generalized (glmer(), binomial family) mixed-effects models, with fixed effects comprising maternal identity, genetic distance and their interaction, and block as a random effect. Model significance was assessed via Type II Wald chi-square ANOVA. To complement these models, we used ggscatter() from the ggpubr package [48] to visualize the relationship between genetic distance and plant performance (total biomass) or survival, separately for each species. This approach included fitting simple linear regression lines and calculating Pearson correlation coefficients with 95% confidence intervals. In parallel, to explore potential variation in iDD among individual trees, we assessed the relationship between maternal identity and genetic distance by faceting the data by maternal source, also on a per-species basis, using the same methodology (Pearson correlation and fitting linear regression lines). All data and code supporting the analyses presented in this study are publicly available in the Dryad Digital Repository [49].

## 3. Results

### (a) Variation in plant survival and performance across species

Patterns of seedling survival varied among species and were significantly influenced by soil inoculum source (maternal tree versus other conspecific) (table 1; *p* = 0.024). Moreover, there was a statistically significant effect of genetic distance between conspecific seed source and conspecific soil source (table 1; *p* = 0.016). *Post hoc* comparisons based on the fitted model revealed that *C. tunu* experienced a significant effect of soil inoculum (figure 3a), with higher predicted survival in soil from non-maternal conspecifics compared to maternal soil (supplementary material, table S4b; Wald *z* = −2.6, odds ratio = 0.33, *p* = 0.009). No significant survival differences were observed within the other species.

**Table 1.** Effect of soil inoculum on seedling survival and performance in eight tropical tree species. We tested whether seedling survival and performance varied with soil inoculum source (maternal tree versus other conspecific tree) and genetic distance (pairwise distance between conspecific adults from which seed and soil inoculum were collected for each pot). Survival was modelled using a generalized linear mixed-effects model (binomial, *n* = 3051 seedlings), and plant performance was analysed using a linear mixed-effects model (*n* = 3051). Seedling performance was quantified as total biomass (above-ground + below-ground biomass). Type II Wald chi-square tests were used to assess fixed effects, with chi-squared values (*χ*²), degrees of freedom (d.f.), and *p*-values reported. Fixed effects with *p* ≤ 0.05 are shown in bold.

response	model	fixed effect	*χ*²^a^	d.f.	*p*
survival	Plant survival ∼ Inoc. Soil * Species + Genetic distance + (1|Adult)+(1|Adult soil)+(1|Block)	Inoculum soil	5.069	1	**0.024**
		Species	320.312	7	**<0.001**
		Genetic distance	5.800	1	**0.016**
		Inoculum soil : Species	5.234	7	0.631
plant performance	Total biomass ∼ Inoc. Soil * Species + Genetic distance+ (1|Adult)+(1|Adult soil) +(1|Block)	Inoculum soil	1.886	1	0.17
		Species	1253.266	7	**<0.001**
		Genetic distance	1.301	1	0.254
		Inoculum soil : Species	14.806	7	**0.039**

^a^Type II Wald chi-square tests.

**Figure 3.**
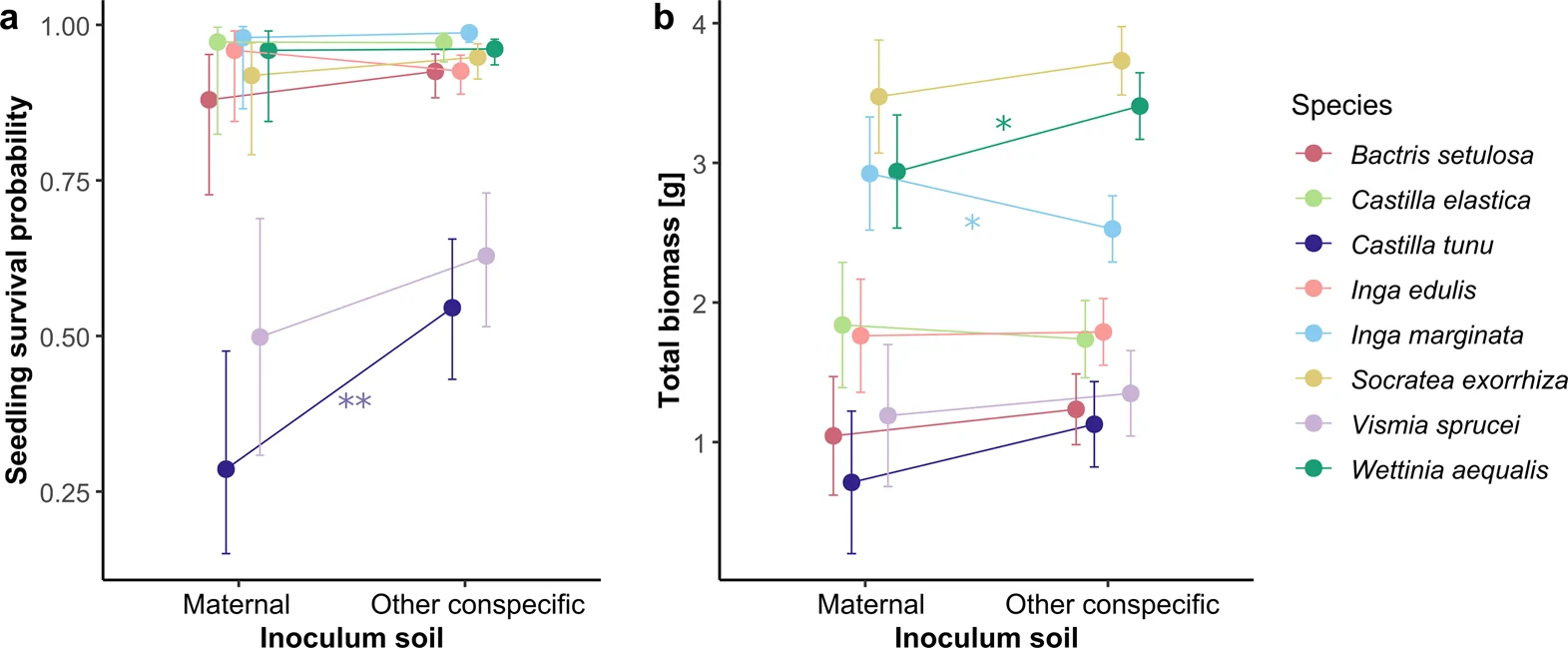
Patterns of seedling survival and plant performance, quantified as total biomass, differed among species growing in maternal versus non-maternal conspecific soil in a shade house fully crossed planting experiment conducted at the FCAT site in Ecuador. (a) Seedling survival rate predicted by a generalized linear mixed-effects model is plotted for each species and treatment group, and (b) predicted seedling performance based on total biomass linear mixed effect models is plotted for each species and treatment group. Error bars on the estimates represent a 95% confidence interval. Asterisks represent the significance level of the *post hoc* test conducted for each species (**p < 0.01, *p < 0.05; supplementary material, table S4b,c).

Patterns of plant performance, quantified as total biomass, also varied among species and reflected species-specific responses to inoculum source, as indicated by a significant species-by-inoculum interaction (table 1; *p* = 0.039), despite the absence of a consistent overall effect of inoculum soil across all species. Genetic distance did not have a significant effect on seedling performance. *Post hoc* comparisons based on model estimates of total biomass revealed species-specific responses (figure 3b), *W. aequalis* exhibited significantly greater total biomass in other conspecific soil compared to maternal soil (supplementary material, table S4c; two-sided *t*-test: *t* = −2.37, estimated difference = −0.469, d.f. = 3048.4, *p* = 0.018), whereas *I. marginata* showed the opposite trend, with significantly greater total biomass in maternal soil (supplementary material, table S4c; two-sided *t*-test: *t* = 2.03, estimated difference = 0.39, d.f. = 3049.6, *p* = 0.043). Other species, including *S. exorrhiza*, *V. sprucei*, *C. tunu* and *B. setulosa*, showed non-significant trends towards greater total biomass in other conspecific soil (supplementary material, table S4c; two-sided *t*-test, *p* > 0.05).

The models presented in table 1 were selected as the best-fitting models to describe the effects of intraspecific plant–soil interactions on plant survival and performance based on AIC. A summary of AIC values for all candidate models is provided in table S4a, supplementary material. To confirm that observed differences in seedling performance were driven by soil inoculum, we compared inoculated treatments (maternal and other conspecific) to sterilized control soil using models that excluded genetic distance as a fixed effect. These results supported the idea that observed effects were driven by soil inoculum, with some exceptions (for detailed information, see supplementary material, S5).

### (b) Within-species variation in plant survival and performance

To further investigate the mechanisms underlying species-specific responses to inoculum source (maternal tree versus non-maternal conspecific) shown in figure 3, we conducted species-level analyses using maternal identity and genetic distance (the pairwise distance between the maternal tree and the conspecific adult tree used as the soil inoculum source) as fixed effects and block as a random effect. For seedling survival, generalized linear mixed-effects models revealed that maternal identity significantly affected outcomes in *B. setulosa* (supplementary material, table S4e; Type II Wald chi square = 17.2, d.f. = 6, *p* = 0.009), *C. tunu* (supplementary material, table S4e; Type II Wald chi square = 16.1, d.f. = 4, *p* = 0.003) and *V. sprucei* (supplementary material, table S4e; Type II Wald chi square = 11.7, d.f. = 4, *p* = 0.02). None of the species showed significant effects of genetic distance or its interaction with maternal identity.

Linear mixed-effects models indicated that for all studied species, total biomass was significantly influenced by maternal identity (supplementary material, table S4d). However, only in *I. marginata* (supplementary material, table S4d; Type II Wald chi-square = 18.59, d.f. = 7, *p* = 0.010) and *V. sprucei* (supplementary material, table S4d; Type II Wald chi-square = 12.31, d.f. = 4, *p* = 0.015) did this effect vary with the genetic distance between seed and inoculum source, as indicated by a significant interaction between maternal identity and genetic distance (adult × genetic distance). For the remaining species, neither genetic distance nor the interaction term was significant.

Figure 4a illustrates species-specific relationships between genetic distance and seedling survival. Scatter plots with fitted linear regressions show that the direction and strength of these relationships vary across species and are not universally significant. Significant negative correlations with genetic distance were observed in *I. edulis* (Pearson correlation, *r* = −0.092, *p* = 0.037) and *I. marginata* (Pearson correlation, *r* = −0.16, *p* < 0.001), showing reduced survival when seedlings were inoculated with soil originating from more genetically distant conspecifics.

**Figure 4.**
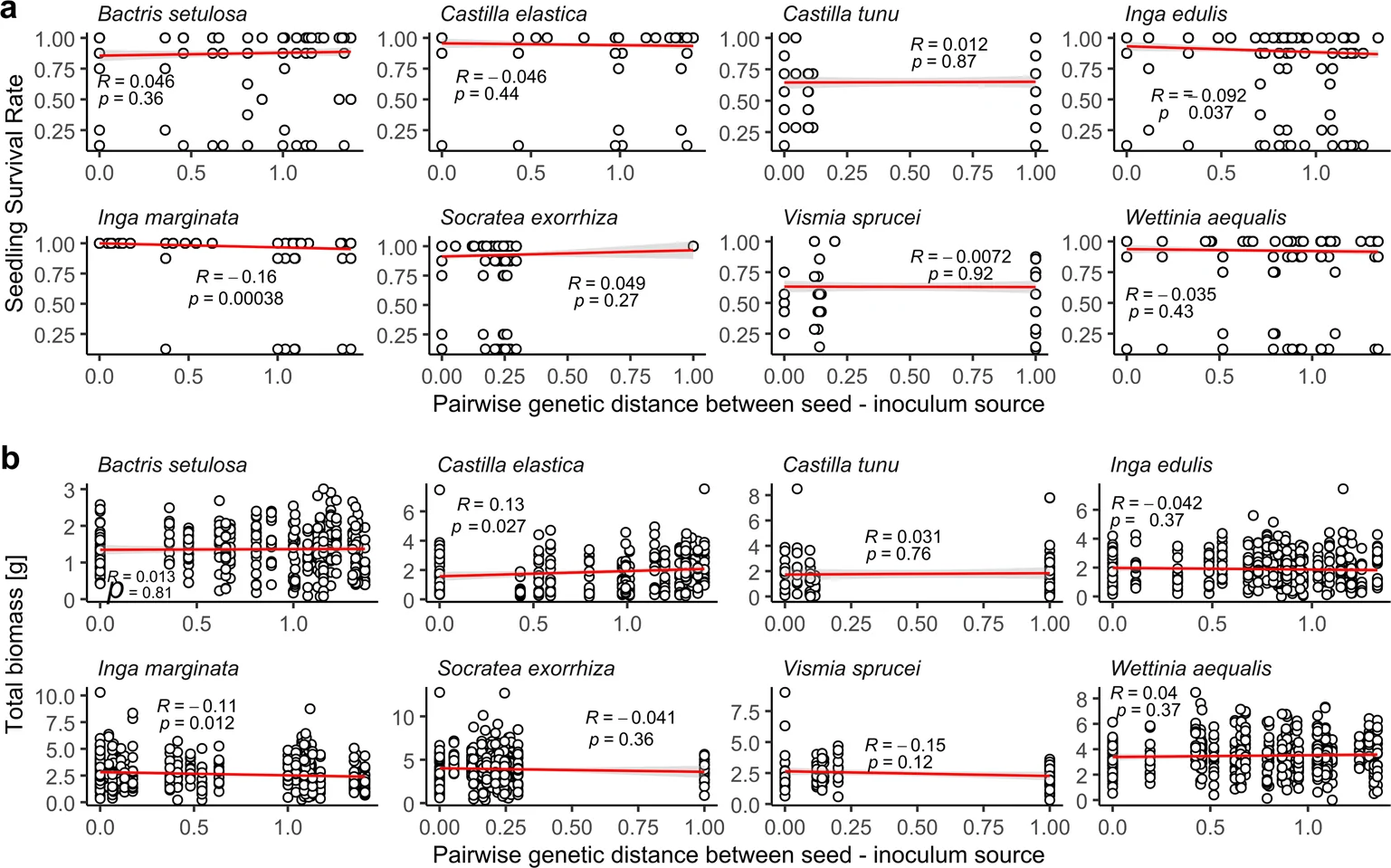
Relationship between seedling responses and the pairwise genetic distance between seed source and soil inoculum source. (a) Seedling survival and (b) seedling performance, quantified as total biomass, show species-specific responses to genetic distance. The direction and strength of these relationships vary among species, indicating that the effects of genetic relatedness between seed and soil inoculum source on plant responses are species-dependent. Pearson correlation coefficients (*R*) and associated *p*-values are shown in each panel.

Figure 4b illustrates species-specific relationships between genetic distance and plant performance, quantified as total biomass. A significant positive correlation was detected in *C. elastica* (Pearson correlation, *r* = 0.13, *p* = 0.027), whereas *I. marginata* again showed a significant negative relationship (Pearson correlation, *r* = −0.11, *p* = 0.012), showing reduced total biomass when seedlings were exposed to soil inoculum from more genetically distant conspecifics. No other species exhibited significant correlations.

To further explore the influence of maternal seed source on seedling responses to genetically distant conspecific soils, scatter plots with fitted linear regressions were generated for each maternal tree within each species (supplementary material, figure S6a–p). These visualizations revealed clear within-species, individual-level variation in plant–soil responses. In some cases, seedlings from particular maternal trees exhibited positive or negative relationships with increasing genetic distance of the inoculum source, whereas seedlings from other conspecifics showed neutral or opposite patterns. This indicates that contrasting intraspecific plant–soil interactions can co-occur within the same species, depending on maternal origin (see supplementary material, S6, for details).

To explore the relationship between genetic variation in the population and iDD across species, we plotted nucleotide diversity (*π*; available for five species) against the species-specific regression slopes shown in figure 4, which describe the relationships between genetic distance and either seedling survival or seedling performance, quantified as total biomass (supplementary material, figure S6q). These slopes were obtained from simple linear regressions fitted separately for each species and were interpreted as indicators of the direction and magnitude of iDD, with more positive slopes indicating stronger negative iDD. We detected a significant positive correlation between nucleotide diversity and the slopes describing seedling performance responses (Pearson correlation: *r* = 0.92, *p* = 0.024; supplementary material, figure S6q(a)), whereas the relationship for seedling survival was positive but non-significant (Pearson correlation: *r* = 0.53, *p* = 0.36; supplementary material, figure S6q(b)). Species exhibiting stronger negative iDD tended to have higher genetic variation (*π*), whereas species showing intraspecific positive iDD, such as *I. marginata*, had lower genetic variation (*π*).

## 4. Discussion

This paper explores the idea that iDD—defined as variation in genotype-specific plant–soil interactions that affect seedling survival or performance either positively or negatively—is potentially an under-appreciated driver of tropical tree diversity. Most previous studies of iDD have focused on a single or a few plant species [10,17,26,27] and used a binary comparison of ‘mother versus other’ when comparing among host individuals. While this work has produced valuable insights, it also raises questions about the ecological significance of genetic relatedness in modulating intraspecific plant–soil interactions, as well as the broader prevalence of iDD dynamics among tropical tree species. Our study suggests that intraspecific variation in plant–soil interactions may be common in tropical forests and that their strength and direction vary among species, and even within a single species among maternal seed sources. Evaluating eight tropical tree species, we observed wide variation among species in both magnitude and direction of these effects, including evidence for both net positive (mutualistic) and net negative (pathogenic) interactions as well as net neutral effects at the species level that may be the consequence of mutualistic and pathogenic interactions effectively balancing each other (see figure 1).

Importantly, seedling survival and growth varied as a function of genetic distance between conspecifics, indicating that plant–soil interactions are sensitive to fine-scale genetic variation within a species. These results show that genetic distance contributes to intraspecific density dependence, by demonstrating that the effects of neighbouring conspecifics are not uniform but depend on their genetic relatedness to the focal individual. In natural systems, seeds are frequently dispersed away from the maternal tree and often germinate nearby other conspecific adults. Our results suggest that the outcome of these interactions may depend on genetic distance, such that seedlings establishing near genetically distant conspecifics may experience different survival and growth outcomes than those near closely related individuals. This highlights genetic distance as a key dimension of intraspecific density dependence, with important implications for the spatial structure and coexistence of tropical tree populations. A key next step will be to test these patterns in field conditions by comparing the fate of seedlings establishing near conspecific adults of varying genetic relatedness with those dispersed away from conspecifics altogether.

### (a) Intraspecific plant–soil interactions are species-specific

The contrasting responses observed across species are consistent with plant–soil interactions mediated by pathogenic or mutualistic microorganisms, or both depending on the species and context. In some species, seedlings grown in soil from genetically distant conspecifics performed better than those in maternal soil, a pattern consistent with negative iDD driven by specialized pathogens. In others, seedlings grown in soil from genetically distant conspecifics showed reduced performance, consistent with positive iDD likely driven by mutualists. In others, there was no net effect consistent with balancing effects of these two opposing processes.

We detected net directional trends of intraspecific plant–soil interactions in three of the eight species studied, *W. aequalis*, *C. tunu* and *I. marginata*, with substantial variation in its incidence and strength across taxa. Previous studies have also reported inconsistent patterns across species. For example, Eck *et al.* [10] found evidence of negative intraspecific plant–microbe interactions in *V. surinamensis* in a shadehouse experiment in Barro Colorado, Panamá, but these patterns were absent in a subsequent field study of the same species and in *Licania panamensis* or *Tachigali panamensis* [11]. Together, these findings suggest that genotype-specific plant–soil interactions reflect species-specific dynamics shaped by plant traits, microbial community composition, environmental context and levels of genetic variation within populations [9,24].

Previous studies have shown that intraspecific genetic diversity can influence plant resistance to soil-borne pathogens [17,26,27]. In our study, genetic diversity (*π*) was positively associated with the strength of negative iDD based on seedling biomass responses among the five species for which this analysis was possible, although the corresponding relationship for seedling survival was not significant. Thus, species with higher genetic diversity may experience stronger negative iDD, but this pattern should be interpreted cautiously given the small number of species included. In principle, negative iDD may occur when soil microbes associated with particular host genotypes reduce the performance of genetically similar seedlings. By limiting the survival or performance of close genetic relatives of nearby conspecifics, these genotype-specific antagonistic interactions could help maintain genetic diversity within populations [10], analogous to how CNDD contributes to the maintenance of species diversity at the community level [2,6,7]. Conversely, positive iDD may arise when soil communities associated with particular genotypes confer a demographic advantage to those genotypes, promoting the recruitment of genetically similar individuals [20,27]. Under such conditions, selection may favour certain genotypes over others, potentially reducing genetic diversity within populations, as suggested by the positive iDD and comparatively low genetic diversity observed in *I. marginata* in our study. However, testing these predictions will require further analyses across a larger number of species.

We minimized abiotic variation by using a standardized, sterilized bulk soil (95% of pot volume), allowing us to attribute seedling responses primarily to differences in soil microbial communities. This was further supported when sterile controls were included in the models for comparison: differences between maternal and other conspecific soils were no longer significant—likely owing to the use of the control treatment as the reference point—but inoculated treatments differed from controls, indicating that survival and performance differences were mediated by soil microbes. However, the loss of significance between maternal and other conspecific soils highlights model limitations detecting fine-scale genetic effects when genetic distance is not accounted for.

### (b) Genetic distance shapes intraspecific plant–soil interactions

The Janzen–Connell hypothesis attributes conspecific negative density dependence (i.e. CNDD) in tropical forests to density- and distance-dependent effects of host-specific natural enemies, particularly soil pathogens [15,50]. Recent studies suggest that evolutionary relationships among neighbouring individuals can also modulate density-dependent dynamics. For instance, Zhu *et al.* [51] documented phylogenetic density dependence across 29 tropical tree species, finding that neighbourhood phylogenetic relatedness negatively affected adult tree survival but positively influenced seedling survival. Building on this perspective, our results reveal that even finer-scale genetic distance among conspecific individuals can influence the strength and direction of plant–soil interactions, thereby shaping density-dependent patterns in seedling performance.

We detected genetic distance-mediated plant–soil interactions in *C. tunu* and *W. aequalis*, where seedling survival or growth increased in soils from genetically distant conspecifics, patterns consistent with pathogen-driven negative iDD. Similar trends have been reported in other systems using genetic or trait dissimilarity measures [17,26,27]. In *C. tunu*, adult trees were spatially aggregated, and nearby adults tended to be genetically similar, suggesting spatial genetic structure that may reflect limited dispersal. However, the seedling response to inoculum from other conspecifics indicates that genetically similar conspecifics may impose stronger negative plant–soil interactions, consistent with negative iDD [27]. By contrast, *W. aequalis* adults occurred within a small spatial radius (approx. 150 m), but genetic distances among adults did not show clear spatial clustering, suggesting weak or absent fine-scale spatial genetic structure [52]. This pattern may be consistent with effective pollen and/or seed dispersal at local scales, as *Wettinia* species are pollinated by specialized insects, *Mystrops* [53], while their seeds are dispersed by mid-sized and large animals [54].

By contrast, *I. marginata* showed the opposite trend, with seedling growth declining as genetic distance increased between seed and soil inoculum sources, consistent with positive iDD potentially driven by mutualistic microbes. Although positive density dependence is rarely reported in tropical forests, previous studies suggest that seedling growth and survival can decline with increasing phylogenetic or genetic distance of conditioning soils, often attributed to variation in mycorrhizal type, nutrient availability and host relatedness [20,26]. While root colonization was not assessed here, *Inga* species commonly associate with arbuscular mycorrhizal fungi [9] and N-fixing bacteria that enhance nutrient uptake and pathogen resistance [55,56], supporting a plausible mutualist-driven positive iDD mechanism in this genus. *I. marginata* showed neither spatial aggregation nor genetic clustering, suggesting extensive seed dispersal or pollen movement within the study area, consistent with evidence that ateline primates are effective seed dispersers for *Inga* and other species [30], among other frugivores.

Our results show that genetic distance among conspecifics modulates both the strength and direction of intraspecific plant–soil interactions, reflecting the interplay of pathogenic and mutualistic microbes. Such interactions may influence not only species coexistence [27] but also the maintenance of genetic diversity within populations as suggested by Eck *et al.* [10], although the relative importance of negative versus positive interactions at the community level remains to be clarified. There is good reason to believe that the outcome of these interactions may be complex and context-dependent; for example, Browne & Karubian [26] observed that positive impacts associated with proximity of seedlings to genetically similar adults early on (<2 yr) shifted to negative impacts as the seedlings aged.

### (c) Within-species variation in plant–soil interactions

While the importance of Janzen–Connell effects on species- and community-level patterns is well established, this and other studies indicate that individual variation may also influence community-level dynamics and coexistence [17,57]. Even at the within-species individual level, we detected consistent variation in the outcome and strength of plant–soil interactions. Maternal seed source significantly affected seedling performance in all species and survival in several species. These patterns indicated that within a species, seedlings can exhibit positive, negative or neutral responses to soil from genetically distant conspecifics depending on the maternal source, as illustrated conceptually in figure 1.

Similar within-species contrasting feedbacks were demonstrated by Schweitzer *et al.* [58] in *Populus angustifolia*, where reciprocal soil transplants among genotypes produced positive, neutral or negative feedbacks depending on maternal origin and soil conditioning. Such contrasting responses within species may explain why the ‘Adult × Genetic distance’ interaction was often non-significant at the species level in our study, given that opposing effects can cancel out when aggregated, preventing the identification of net outcomes. Mathematical models support the idea that positive, neutral and negative interactions can arise within a species depending on the degree of genetic difference between seed and soil sources and the strength of environmental gradients [59]. Also, empirical evidence for tree-to-tree variation in soil biotic interactions comes from temperate species such as *Prunus serotina* [57] and crops like *Medicago sativa*, where differences among maternal lines relate to plant traits [23]. In our study, the consistent influence of maternal seed source on seedling responses, combined with the controlled soil conditions, suggests that genetic variation among conspecific adults is a plausible contributor to the observed patterns.

Both positive and negative within-species plant–soil interactions can influence genetic diversity and seedling diversity: negative interactions may promote diversity by limiting recruitment of closely related individuals, whereas positive interactions may reduce it by favouring certain seed sources. Because beneficial and antagonistic microbes can act simultaneously, the net effect depends on their relative strengths [9,20,23]. Many previous studies seeking to detect genotype-specific effects have used relatively coarse contrasts such as maternal versus non-maternal soils [11,60]. Our findings indicate that such designs may overlook fine-scale genetic variation when responses differ among maternal sources. We therefore advocate for experimental designs that explicitly incorporate a metric of genetic relatedness to account for genetic variation within species to better capture the complexity of plant–soil interactions in tropical forests.

Overall, these results highlight the ecological significance of genetic variation in modulating plant–soil interactions within tropical tree species. They demonstrate that both pathogenic and mutualistic microorganisms can contribute to intraspecific density dependence and that these processes can operate simultaneously within a species, with potentially significant consequences for species coexistence and community dynamics that require further investigation.

## Supplementary Material



## Data Availability

The data and code supporting this study are publicly available from Dryad [49]. DNA sequence data are available in the NCBI Sequence Read Archive (SRA) under BioProject accession PRJNA1363971. Supplementary material is available online [61].
