# Supplementary material for: The role of genetic distance in modulating intraspecific plant–soil interactions among a diverse assemblage of tropical tree species seedlings
Source: Proc Biol Sci. 2026 Oct 21;293(2081):20253199. doi: 10.1098/rspb.2025.3199 (PMC13682881; doi:10.1098/rspb.2025.3199)
Supplement: Supplementary file 1 [file rspb.2025.3199.zip › rspb.20253199/rspb20253199_si_001.docx]

**Electronic Supplementary Material**

## **The role of genetic distance in modulating intraspecific plant–soil interactions among a diverse assemblage of tropical tree species seedlings**

Rosa I. Soria^1^, Karla Zambrano^2^, Carlos Aulestia^2^, Gonzalo Rivas-Torres^3^, Jordan Karubian^1,2,3^ and Sunshine Van Bael^1^

^1^ Department of Ecology and Evolutionary Biology, Tulane University, New Orleans, USA

^2^ Fundación para la Conservación de los Andes Tropicales (FCAT), Quinindé, Ecuador

^3^ Universidad San Francisco de Quito, Estación de Biodiversidad Tiputini, Colegio de Ciencias Biológicas y Ambientales, Quito, Ecuador

Corresponding author: [rsoriapenafiel@tulane.edu](mailto:rsoriapenafiel@tulane.edu)

Journal: Proceedings of the Royal Society B

Article DOI: <https://doi.org/10.1098/rspb.2025.3199>

**INDEX:**

**Supplementary 1:** Details of experimental design

**Supplementary 2:** Estimating pairwise genetic distances among conspecific adults

**Supplementary 3:** Evaluating the relationship between geographic distance and genetic distance

**Supplementary 4:** Evaluating model fit for seedling survival and performance

**Supplementary 5:** Evaluating treatment effects with control treatments (autoclaved soil) as reference

**Supplementary 6:** Effect of maternal source on survival and performance

### Supplementary 1: Details of experimental design

**Supplementary Table S1a: Summary of the experimental design for the crossed planting experiment.** The experiment included 8 tropical tree species in which seeds were inoculated with soil from their maternal tree or with live soil inoculum from other conspecifics or had no inoculum (control).

| Scientific name | Family | Number of adults^a^ | Factorial design^b^ | Total number of seedlings |
| --- | --- | --- | --- | --- |
| *Inga marginata* | Fabaceae | 8 | 8 × 9 × 8 | 576 |
| *Castilla tunu* | Moraceae | 5 | 5 × 6 × 8 | 240 |
| *Wettinia aequalis* | Arecaceae | 8 | 8 ×9 × 8 | 576 |
| *Inga edulis* | Fabaceae | 8 | 8 × 9 × 8 | 576 |
| *Castilla elastica* | Moraceae | 6 | 6 × 7 × 8 | 336 |
| *Vismia sprucei* | Hypericaceae | 5 | 5 × 6 × 8 | 240 |
| *Bactris setulosa* | Arecaceae | 8 | 8 × 9 × 8 | 576 |
| *Socratea exorrhiza* | Arecaceae | 7 | 7 × 8 × 8 | 448 |

^a^This is the number of adult trees from each species from which seeds and live soil inoculum were collected for the fully crossed planting experiment.

^b^This represents number of adult trees $\times$ number of sources of soil inoculum (adult trees + control) $\times\text{ }$ replicates

### Supplementary 2: Estimating pairwise genetic distances among conspecific adults

**Supplementary Table S2a: Optimization of three core STACKS parameters, -m, -M, and -n, and the associated measures of genetic diversity for each combination of parameters**. Bold letters show the optimal run parameters chosen for each species.

| **Species** |  | **Parameters tested** | | | **Variant positions (avg over populations)** | | | | **All positions (avg over pops)** |
| --- | --- | --- | --- | --- | --- | --- | --- | --- | --- |
| *Bactris*  *setulosa* | Name | -m | -M | n | *He* | SE | Pi (*π*) | SE | Polymorph sites |
|  | Opt1 | 3 | 1 | 1 | 0.015 | 0.101 | 0.006 | 0.374 | 455 |
|  | Opt2 | 3 | 2 | 2 | 0.287 | 0.013 | 0.417 | 0.006 | 560 |
|  | **Opt3** | **3** | **3** | **3** | **0.291** | **0.012** | **0.414** | **0.006** | **582** |
|  | Opt4 | 3 | 4 | 4 | 0.298 | 0.012 | 0.415 | 0.006 | 609 |
|  | Opt5 | 3 | 5 | 5 | 0.290 | 0.011 | 0.413 | 0.005 | 629 |
|  | Opt6 | 3 | 6 | 6 | 0.288 | 0.011 | 0.412 | 0.005 | 639 |
|  | Opt7 | 3 | 7 | 7 | 0.292 | 0.011 | 0.413 | 0.005 | 651 |
| *Castilla*  *elastica* | Opt1 | 2 | 1 | 1 | 0.191 | 0.037 | 0.554 | 0.012 | 75 |
|  | Opt2 | 2 | 2 | 2 | 0.289 | 0.034 | 0.545 | 0.010 | 121 |
|  | Opt3 | 2 | 3 | 3 | 0.180 | 0.028 | 0.567 | 0.010 | 125 |
|  | Opt4 | 2 | 2 | 1 | 0.293 | 0.039 | 0.555 | 0.010 | 99 |
|  | **Opt5** | **2** | **2** | **3** | **0.237** | **0.028** | **0.537** | **0.010** | **156** |
|  | Opt6 | 2 | 1 | 3 | 0.144 | 0.024 | 0.545 | 0.011 | 142 |
| *Inga*  *marginata* | Opt1 | 3 | 1 | 1 | 0.300 | 0.003 | 0.293 | 0.002 | 6397 |
|  | **Opt2** | **3** | **2** | **2** | **0.303** | **0.003** | **0.297** | **0.002** | **6543** |
|  | Opt3 | 3 | 3 | 3 | 0.304 | 0.003 | 0.300 | 0.002 | 6502 |
|  | Opt4 | 3 | 4 | 4 | 0.302 | 0.003 | 0.300 | 0.002 | 6434 |
| *Inga*  *edulis* | Opt1 | 3 | 1 | 1 | 0.256 | 0.004 | 0.280 | 0.003 | 2201 |
|  | Opt2 | 3 | 2 | 2 | 0.260 | 0.004 | 0.284 | 0.003 | 2509 |
|  | **Opt3** | **3** | **3** | **3** | **0.263** | **0.004** | **0.286** | **0.003** | **2612** |
|  | Opt4 | 3 | 4 | 4 | 0.266 | 0.004 | 0.287 | 0.003 | 2649 |
| *Wettinia*  *aequalis* | Opt1 | 3 | 1 | 1 | 0.377 | 0.011 | 0.399 | 0.005 | 646 |
|  | **Opt2** | **3** | **2** | **2** | **0.375** | **0.012** | **0.398** | **0.005** | **696** |
|  | Opt3 | 3 | 3 | 3 | 0.361 | 0.012 | 0.398 | 0.005 | 682 |
|  | Opt4 | 3 | 4 | 4 | 0.372 | 0.012 | 0.410 | 0.005 | 696 |

**Supplementary Table S2b: Summary of ddRadSeq data coverage and approach used for estimating genetic distances.**

| **Species** | **Depth of coverage** | | **Approach used for genetic distance estimation** |
| --- | --- | --- | --- |
|  | Min | Max |  |
| *Bactris setulosa* | 7.07 | 14.84 | Stacks/Populations (PCA-based) |
| *Castilla elastica* | 2.98 | 4.43 | Stacks/Populations (PCA-based) |
| *Inga marginata* | 8.06 | 10.67 | Stacks/Populations (PCA-based) |
| *Inga edulis* | 7.24 | 11.97 | Stacks/Populations (PCA-based) |
| *Wettinia aequalis* | 7.19 | 20.11 | Stacks/Populations (PCA-based) |
| *Vismia sprucei* | 2.21 | 3.90 | Mash (k-mer–based) |
| *Socratea exorrhiza* | 2.08 | 3.10 | Mash (k-mer–based) |
| *Castilla tunu* | 3.40 | 8.60 | Mash (k-mer–based) |

**Supplementary Figure S2a: Principal component analysis of genotypic variation among *Bactris setulosa* individuals based on ddRADseq SNPs analysis using optimized STACKS parameters.**

**Supplementary Figure S2b: Principal component analysis of genotypic variation among *Castilla elastica* individuals based on ddRADseq SNPs using optimized STACKS parameters.**

**Supplementary Figure S2c: Principal component analysis of genotypic variation among *Inga edulis* individuals based on ddRADseq SNPs using optimized STACKS parameters.**

**Supplementary Figure S2d: Principal component analysis of genotypic variation among *Inga marginata* individuals based on ddRADseq SNPs using optimized STACKS parameters.**

**Supplementary Figure S2e: Principal component analysis of genotypic variation among *Wettinia aequalis* individuals based on ddRADseq SNPs using optimized STACKS parameters.**

### Supplementary 3: Evaluating the relationship between geographic distance and genetic distance

The relationship between geographic and genetic distance was visualized separately for each species using the ggscatter() function in the ggpubr package (1). Pearson correlation coefficients and fitted linear regression lines were used to assess the association between both variables. As expected, a significant positive relationship was found for seven of the eight species (Supplementary 3: Figure S3a), indicating that genetic distance generally increased with geographic distance among conspecific adults. However, because the primary objective of this study was to examine how genetic distance between conspecifics influences intraspecific plant-soil interactions, all subsequent statistical analyses focused exclusively on genetic distance.

**Supplementary Figure S3a: Relationship between geographic distance and genetic distance between conspecific adults.** Genetic distance increases with geographic distance showing a positive significant linear relationship for 7 out of the 8 species studied. Genetic distance is the pairwise distance estimated between conspecific adults from where a seed and soil inoculum were collected. The red line is the linear regression line and the grey areas represent the 95% confidence interval. Pearson correlation coefficients (*R*) and associated *p*-values are shown in each panel.

### Supplementary 4: Evaluating model fit for survival and performance

**Supplementary Table** **S4a:** Model comparison assessing seedling survival and plant performance as a function of soil inoculum type (maternal vs. other conspecific), species identity, and pairwise genetic distance between conspecific adults. Models were evaluated using Akaike’s information criterion (AIC). All models included random intercepts for maternal tree (Adult), soil donor tree (Adult soil), and experimental block (Block). The selected model for each response variable is highlighted in bold.

| **Response** | **Model** | **d.f.** | **AIC** | **Delta AIC** |
| --- | --- | --- | --- | --- |
| Survival | **Survival ~ Inoc. Soil * Species + Genetic distance + (1\|Adult) + (1\|Adult soil) + (1\|Block)** | **20** | **1,567.588** | **0.000** |
|  | Survival ~ Inoc. Soil * Species * Genetic distance + (1\|Adult) + (1\|Adult soil) + (1\|Block) | 27 | 1,579.313 | 11.726 |
|  | Survival ~ Inoc. Soil + Species * Genetic distance + (1\|Adult) + (1\|Adult soil) + (1\|Block) | 20 | 1,571.317 | 3.729 |
| Plant Performance | **Total biomass ~ Inoc. Soil * Species + Genetic distance+ (1\|Adult)+(1\|Adult soil) +(1\|Block)** | **21** | **10,774.76** | **3.794** |
|  | Total biomass ~ Inoc. Soil + Species * Genetic distance + (1\|Adult)+(1\|Adult soil) +(1\|Block) | 21 | 10,781.38 | 10.414 |
|  | Total biomass ~ Inoc. Soil * Species + (1\|Adult)+(1\|Adult soil) +(1\|Block) | 20 | 10,770.97 | 0.000 |

For plant performance we selected the model with the lowest AIC that specifically tested for genetic distance as this was the core question of this study.

**Supplementary Table S4b: Pairwise contrasts of soil inoculum source (maternal vs. other conspecific) on seedling survival within each species.** Post hoc comparisons were based on estimated marginal means (EMMs) from the generalized linear mixed-effects model (GLMM from **Table 2**) using Wald *z*-tests. Comparisons were performed with the emmeans package (type = "response"), and provide species-specific contrasts in predicted survival probability. The odds ratio, standard error, z-ratio, and *p*-value are reported for each species-level contrast.

| **Contrast** | **Species** | **odds.ratio** | **SE** | **d.f.** | **null** | ***z*.ratio** | ***p*** |
| --- | --- | --- | --- | --- | --- | --- | --- |
| Maternal *vs* Other conspecific | *Bactris setulosa* | 0.591 | 0.329 | Inf | 1 | -0.946 | 0.344 |
|  | *Castilla elastica* | 1.038 | 1.134 | Inf | 1 | 0.035 | 0.972 |
|  | *Castilla tunu* | 0.334 | 0.140 | Inf | 1 | -2.622 | **0.009** |
|  | *Inga edulis* | 1.881 | 1.439 | Inf | 1 | 0.826 | 0.409 |
|  | *Inga marginata* | 0.612 | 0.670 | Inf | 1 | -0.449 | 0.654 |
|  | *Socratea exorrhiza* | 0.618 | 0.351 | Inf | 1 | -0.849 | 0.396 |
|  | *Vismia sprucei* | 0.587 | 0.242 | Inf | 1 | -1.291 | 0.197 |
|  | *Wettinia aequalis* | 0.945 | 0.736 | Inf | 1 | -0.073 | 0.942 |

**Supplementary Table S4c: Pairwise contrasts of soil inoculum source (maternal vs. other conspecific) on plant performance within each species.** Post hoc comparisons were based on estimated marginal means (EMMs) from the linear mixed-effects model (LMM from **Table 2**) using a two-sided t-test with Satterthwaite-approximated degrees of freedom. Pairwise comparisons were extracted using the emmeans package (type = "response"), and provide species-specific contrasts in predicted plant performance. The estimated difference, standard error, degrees of freedom, t-ratio and *p*-value are reported for each species-level contrast.

| **contrast** | **Species** | **Estimate** | **SE** | **d.f.** | **t.ratio** | ***p*** |
| --- | --- | --- | --- | --- | --- | --- |
| Maternal *vs* Other conspecific | *Bactris setulosa* | -0.190 | 0.214 | 3,048.298 | -0.888 | 0.375 |
|  | *Castilla elastica* | 0.101 | 0.235 | 3,048.183 | 0.432 | 0.666 |
|  | *Castilla tunu* | -0.416 | 0.265 | 3,046.609 | -1.573 | 0.116 |
|  | *Inga edulis* | -0.028 | 0.198 | 3,048.253 | -0.139 | 0.890 |
|  | *Inga marginata* | 0.397 | 0.196 | 3,049.597 | 2.027 | **0.043** |
|  | *Socratea exorrhiza* | -0.256 | 0.184 | 3,042.449 | -1.390 | 0.165 |
|  | *Vismia sprucei* | -0.159 | 0.264 | 3,045.695 | -0.604 | 0.546 |
|  | *Wettinia aequalis* | -0.469 | 0.198 | 3,048.379 | -2.367 | **0.018** |

**Supplementary Table S4d: Mixed linear effect model results assessing the effect of maternal identity (Adult) and genetic distance between conspecific adults on plant performance**

| **Species** | **Model** | **Term** | ***χ*²*** | **d.f.** | ***p*.value** |
| --- | --- | --- | --- | --- | --- |
| *Bactris setulosa* | Total biomass ~ Adult * Genetic distance + (1\|Block) | Adult | 112.040 | 6 | **< 0.001** |
|  |  | Genetic distance | 0.163 | 1 | 0.687 |
|  |  | Adult:Genetic distance | 1.778 | 6 | 0.939 |
| *Castilla elastica* | Total biomass ~ Adult * Genetic distance + (1\|Block) | Adult | 174.817 | 5 | **< 0.001** |
|  |  | Genetic distance | 1.611 | 1 | 0.204 |
|  |  | Adult:Genetic distance | 8.211 | 1 | 0.145 |
| *Castilla tunu* | Total biomass ~ Adult * Genetic distance + (1\|Block) | Adult | 27.234 | 4 | **< 0.001** |
|  |  | Genetic distance | 1.116 | 1 | 0.291 |
|  |  | Adult:Genetic distance | 6.412 | 4 | 0.17 |
| *Inga marginata* | Total biomass ~ Adult * Genetic distance + (1\|Block) | Adult | 15.172 | 7 | **0.034** |
|  |  | Genetic distance | 7.753 | 1 | **0.005** |
|  |  | Adult:Genetic distance | 18.592 | 7 | **0.01** |
| *Inga edulis* | Total biomass ~ Adult + Genetic distance + (1\|Block) | Adult | 62.502 | 7 | **< 0.001** |
|  |  | Genetic distance | 0.256 | 1 | 0.613 |
| *Socratea exorrhiza* | Total biomass ~ Adult * Genetic distance + (1\|Block) | Adult | 116.782 | 7 | **< 0.001** |
|  |  | Genetic distance | 0.003 | 1 | 0.958 |
|  |  | Adult:Genetic distance | 8.559 | 7 | 0.286 |
| *Vismia sprucei* | Total biomass ~ Adult * Genetic distance + (1\|Block) | Adult | 19.273 | 4 | **0.001** |
|  |  | Genetic distance | 0.870 | 1 | 0.351 |
|  |  | Adult:Genetic distance | 12.305 | 4 | **0.015** |
| *Wettinia aequalis* | Total biomass ~ Adult * Genetic distance + (1\|Block) | Adult | 129.657 | 7 | **< 0.001** |
|  |  | Genetic distance | 1.087 | 1 | 0.297 |
|  |  | Adult:Genetic distance | 9.515 | 7 | 0.218 |

* *Type II Wald chisquare test*

**Note:** For each species, models with and without the interaction between maternal identity (Adult) and genetic distance were tested, and the model shown corresponds to the best-supported model according to AIC. Therefore, interaction terms are reported only for species in which the interaction model was selected. For Inga edulis, the best-supported model for total biomass did not include the interaction term.

**Supplementary Table S4e: Results from the generalized linear mixed-effects model assessing the effects of maternal identity (Adult) and genetic distance on seedling survival.** Genetic distance refers to the pairwise distance between the conspecific adult tree from which seeds were collected and the conspecific tree from which soil inoculum was sourced.

| **Species** | **Model** | **Term** | ***χ*²*** | **d.f.** | ***p*.value** |
| --- | --- | --- | --- | --- | --- |
| *Bactris setulosa* | Survival ~ Adult*Genetic distance + (1\|Block) | Adult | 17.187 | 6 | **0.009** |
|  |  | Genetic distance | 0.000 | 1 | 0.983 |
|  |  | Adult:Genetic distance | 7.845 | 6 | 0.25 |
| *Castilla elastica* | Survival ~ Adult*Genetic distance + (1\|Block) | Adult | 2.637 | 5 | 0.756 |
|  |  | Genetic distance | 0.877 | 1 | 0.349 |
|  |  | Adult:Genetic distance | 0.683 | 5 | 0.984 |
| *Castilla tunu* | Survival ~ Adult*Genetic distance + (1\|Block) | Adult | 16.183 | 4 | **0.003** |
|  |  | Genetic distance | 0.154 | 1 | 0.694 |
|  |  | Adult:Genetic distance | 3.341 | 4 | 0.502 |
| *Inga marginata* | Survival ~ Adult*Genetic distance + (1\|Block) | Adult | 2.521 | 7 | 0.925 |
|  |  | Genetic distance | 1.339 | 1 | 0.247 |
|  |  | Adult:Genetic distance | 1.484 | 7 | 0.983 |
| *Inga edulis* | Survival ~ Adult*Genetic distance + (1\|Block) | Adult | 10.097 | 7 | 0.183 |
|  |  | Genetic distance | 0.551 | 1 | 0.458 |
|  |  | Adult:Genetic distance | 6.914 | 7 | 0.438 |
| *Socratea exorrhiza* | Survival ~ Adult*Genetic distance + (1\|Block) | Adult | 10.529 | 7 | 0.161 |
|  |  | Genetic distance | 1.235 | 1 | 0.266 |
|  |  | Adult:Genetic distance | 1.701 | 7 | 0.975 |
| *Vismia sprucei* | Survival ~ Adult*Genetic distance + (1\|Block) | Adult | 11.710 | 4 | **0.020** |
|  |  | Genetic distance | 0.069 | 1 | 0.793 |
|  |  | Adult:Genetic distance | 7.471 | 4 | 0.113 |
| *Wettinia aequalis* | Survival ~ Adult*Genetic distance + (1\|Block) | Adult | 4.947 | 7 | 0.666 |
|  |  | Genetic distance | 0.181 | 1 | 0.671 |
|  |  | Adult:Genetic distance | 3.366 | 7 | 0.849 |

* *Type II Wald chisquare tests*

**Supplementary Figure S4a: Marginal effects of fixed terms included in the model explaining seedling survival (see Table 2 main manuscript).** (a) Marginal effect of genetic distance on seedling survival across all studied species, showing that survival tended to decrease with increasing genetic distance between the maternal seed source and soil inoculum source. (b) Effect of the interaction between inoculum soil and species, indicating that *Vismia sprucei* and *Castilla tunu* tended to have higher predicted survival probabilities in non-maternal conspecific soils than in their maternal soils.

### Supplementary 5: Evaluating treatment effects with control soil as reference

As shown in **Supplementary Figure S5a**, *Castilla tunu* showed no significant differences in survival probability or performance between control and maternal soil. However, seedlings exhibited significantly higher survival in the other conspecific soil treatment compared to the control (Table S5a; Wald *z* = –4.56, odds ratio = 0.139, *p* < 0.001), suggesting intraspecific plant-soil effects. Similar patterns were observed for predicted Total biomass in *Castilla tunu* (Table S5b; *t* = –3.98, estimated difference = –0.78, ,d.f. = 75.60, *p* = 0.002). In contrast, *Inga marginata* exhibited significantly higher performance in maternal soil compared to control (Table S5b; *t* = –5.99, estimated difference = –1.36, d.f. = 87.99, *p* < 0.001), and in other conspecific soil relative to control (Table S5b; *t* = –5.36, estimated difference = –0.88, d.f. = 25.12, *p* < 0.001). Additionally, performance was significantly higher in other conspecific soil compared to maternal soil (Table S5b; *t* = 2.64, estimated difference = 0.48, d.f. = 4029.01, *p* = 0.022), indicating positive intraspecific plant-soil interactions.

**Supplementary Figure S5a: Patterns of seedling survival and Total biomass growing in control (autoclaved), maternal soil and other conspecific soil varied among species in a shade house reciprocal planting experiment in FCAT site in Ecuador.** (a) Seedling survival probability predicted by a generalized linear mixed‐effects model is plotted for each species and treatment group, and (b) Total biomass predicted from total biomass using linear mixed effect models is plotted for each species and treatment group. Error bars on the estimates represent a 95% confidence interval. Asterisks represent the significance level of the post hoc test conducted for each species (***= *p* < 0.001, ** = *p* < 0.01, * = *p* < 0.05).

**Supplementary Table S5a: Pairwise contrasts of the model that included control soil to assess the impact of inoculum source (control, maternal or other conspecific) on seedling survival within each species.** Post hoc comparisons were based on estimated marginal means (EMMs) from the generalized linear mixed-effects model (Survival ~ Inoc. Soil * Species + (1|Adult) + (1|Adult soil) + (1|Block)) using Wald *z*-tests. Comparisons were performed with the emmeans package (type = "response"), and provide species-specific contrasts in predicted survival probability. The odds ratio, standard error, *z*-ratio, and *p*-value are reported for each species-level contrast.

| **Contrast** | **Species** | **odds.ratio** | **SE** | **d.f.** | **null** | ***z*.ratio** | ***p*.value** |
| --- | --- | --- | --- | --- | --- | --- | --- |
| Control - Maternal | *Bactris setulosa* | 0.580 | 0.334 | Inf | 1 | -0.947 | 0.611 |
| Control - Other conspec | *Bactris setulosa* | 0.830 | 0.307 | Inf | 1 | -0.503 | 0.870 |
| Maternal - Other cons | *Bactris setulosa* | 1.431 | 0.702 | Inf | 1 | 0.730 | 0.746 |
| Control - Maternal | *Castilla elastica* | 0.358 | 0.404 | Inf | 1 | -0.911 | 0.633 |
| Control - Other conspec | *Castilla elastica* | 0.678 | 0.412 | Inf | 1 | -0.639 | 0.798 |
| Maternal - Other conspec | *Castilla elastica* | 1.894 | 2.014 | Inf | 1 | 0.601 | 0.820 |
| Control - Maternal | *Castilla tunu* | 0.296 | 0.156 | Inf | 1 | -2.316 | 0.054 |
| Control - Other conspec | *Castilla tunu* | 0.141 | 0.061 | Inf | 1 | -4.556 | **0.000** |
| Maternal - Other conspec | *Castilla tunu* | 0.477 | 0.186 | Inf | 1 | -1.899 | 0.139 |
| Control - Maternal | *Inga edulis* | 0.472 | 0.392 | Inf | 1 | -0.903 | 0.638 |
| Control - Other conspec | *Inga edulis* | 1.459 | 0.657 | Inf | 1 | 0.838 | 0.680 |
| Maternal - Other conspec | *Inga edulis* | 3.087 | 2.273 | Inf | 1 | 1.531 | 0.276 |
| Control - Maternal | *Inga marginata* | 0.481 | 0.549 | Inf | 1 | -0.641 | 0.797 |
| Control - Other conspec | *Inga marginata* | 0.485 | 0.323 | Inf | 1 | -1.087 | 0.522 |
| Maternal - Other conspec | *Inga marginata* | 1.009 | 1.083 | Inf | 1 | 0.008 | 1.000 |
| Control - Maternal | *Socratea exorrhiza* | 1.613 | 1.160 | Inf | 1 | 0.665 | 0.784 |
| Control - Other conspec | *Socratea exorrhiza* | 1.136 | 0.624 | Inf | 1 | 0.233 | 0.971 |
| Maternal - Other conspec | *Socratea exorrhiza* | 0.705 | 0.398 | Inf | 1 | -0.619 | 0.809 |
| Control - Maternal | *Vismia sprucei* | 0.622 | 0.294 | Inf | 1 | -1.004 | 0.574 |
| Control - Other conspec | *Vismia sprucei* | 0.503 | 0.185 | Inf | 1 | -1.873 | 0.147 |
| Maternal - Other conspec | *Vismia sprucei* | 0.809 | 0.315 | Inf | 1 | -0.544 | 0.849 |
| Control - Maternal | *Wettinia aequalis* | 0.777 | 0.679 | Inf | 1 | -0.289 | 0.955 |
| Control - Other conspec | *Wettinia aequalis* | 1.196 | 0.655 | Inf | 1 | 0.328 | 0.943 |
| Maternal - Other conspec | *Wettinia aequalis* | 1.541 | 1.157 | Inf | 1 | 0.575 | 0.833 |

**Supplementary Table S5b: Pairwise contrasts of the model that included control soil to assess the impact of inoculum source (control, maternal or other conspecific) on total biomass within each species.** Post hoc comparisons were based on estimated marginal means (EMMs) from the linear mixed-effects model (Total biomass ~ Inoc. Soil*Species + (1|Adult)+(1|Adult soil) +(1|Block)) using a two-sided *t*-test with Satterthwaite-approximated degrees of freedom. Pairwise comparisons were extracted using the emmeans package (type = "response"), and provide species-specific contrasts in predicted Total biomass. The estimated difference, standard error, degrees of freedom, t-ratio and P-value are reported for each species-level contrast.

| **Contrast** | **Species** | **Estimate** | **SE** | **d.f.** | ***t*.ratio** | ***p*** |
| --- | --- | --- | --- | --- | --- | --- |
| Control – Maternal | *Bactris setulosa* | 0.056 | 0.227 | 87.999 | 0.248 | 0.967 |
| Control – Other conspec | *Bactris setulosa* | -0.011 | 0.166 | 25.121 | -0.066 | 0.998 |
| Maternal – Other conspec | *Bactris setulosa* | -0.068 | 0.180 | 4,029.007 | -0.376 | 0.925 |
| Control – Maternal | *Castilla elastica* | -0.456 | 0.257 | 140.260 | -1.773 | 0.182 |
| Control – Other conspec | *Castilla elastica* | -0.253 | 0.189 | 42.115 | -1.333 | 0.385 |
| Maternal – Other conspec | *Castilla elastica* | 0.203 | 0.213 | 4,029.007 | 0.955 | 0.605 |
| Control – Maternal | *Castilla tunu* | -0.434 | 0.295 | 237.702 | -1.470 | 0.307 |
| Control – Other conspec | *Castilla tunu* | -0.788 | 0.220 | 75.604 | -3.583 | **0.002** |
| Maternal – Other conspec | *Castilla tunu* | -0.354 | 0.254 | 4,029.007 | -1.394 | 0.344 |
| Control – Maternal | *Inga edulis* | -0.112 | 0.227 | 87.999 | -0.494 | 0.874 |
| Control – Other conspec | *Inga edulis* | -0.054 | 0.166 | 25.121 | -0.326 | 0.943 |
| Maternal – Other conspec | *Inga edulis* | 0.058 | 0.180 | 4,029.007 | 0.324 | 0.944 |
| Control – Maternal | *Inga marginata* | -1.364 | 0.227 | 87.999 | -5.994 | **0.000** |
| Control – Other conspec | *Inga marginata* | -0.888 | 0.166 | 25.121 | -5.356 | **0.000** |
| Maternal – Other conspec | *Inga marginata* | 0.475 | 0.180 | 4,029.007 | 2.643 | **0.022** |
| Control – Maternal | *Socratea exorrhiza* | 0.261 | 0.227 | 87.999 | 1.149 | 0.487 |
| Control – Other conspec | *Socratea exorrhiza* | 0.029 | 0.166 | 25.121 | 0.177 | 0.983 |
| Maternal – Other conspec | *Socratea exorrhiza* | -0.232 | 0.180 | 4,029.007 | -1.291 | 0.400 |
| Control – Maternal | *Vismia sprucei* | -0.404 | 0.295 | 237.507 | -1.367 | 0.360 |
| Control – Other conspec | *Vismia sprucei* | -0.507 | 0.220 | 75.549 | -2.304 | 0.061 |
| Maternal – Other conspec | *Vismia sprucei* | -0.103 | 0.254 | 4,029.007 | -0.407 | 0.913 |
| Control – Maternal | *Wettinia aequalis* | 0.429 | 0.227 | 87.999 | 1.885 | 0.149 |
| Control – Other conspec | *Wettinia aequalis* | 0.045 | 0.166 | 25.121 | 0.273 | 0.960 |
| Maternal – Other conspec | *Wettinia aequalis* | -0.383 | 0.180 | 4,029.007 | -2.133 | 0.083 |

### Supplementary 6: Effect of maternal source on survival and performance

To further examine the role of maternal seed source (adult) in modulating seedling responses to genetically distant conspecific soil, scatterplots with fitted linear regressions were generated for each adult within each species (Figures S6a–S6p). These visualizations revealed within-species, individual-level variation in soil feedback responses. In some cases, seedlings from particular maternal trees exhibited positive or negative responses to increasing genetic distance of the inoculum source, while seedlings from other conspecifics showed opposite or neutral responses. For example, in *Castilla elastica* (Figure S6b), seedlings from adult 4 tended to have lower total biomass as genetic distance between maternal and inoculum sources increased (Pearson correlation, *r* = –0.37, *p* = 0.009), whereas seedlings from other adults showed no significant relationship, as indicated by near-horizontal regression lines, suggesting neutral soil feedbacks. Similarly, in *Vismia sprucei* (Figure S5g), seedlings from adult 4 displayed a significant positive relationship with genetic distance (Pearson correlation: *r* = 0.48, *p* < 0.05), indicating improved total biomass with more genetically distant soil inoculum while other adults showed no significant relationship between total biomass and genetic distance.

In *Inga marginata* (Figure S6e), seedlings from adults 2, 5, and 7 exhibited significantly negative correlations between genetic distance and performance (Pearson correlations: *r* = –0.35, *p* = 0.0049; *r* = –0.25, *p* = 0.043; *r* = –0.39, *p* < 0.001), while other maternal lines showed no significant trend. Similar patterns were observed in *Socratea exorrhiza* and *Wettinia aequalis* (Figure S5f and Figure S5h), reinforcing the presence of individual-level variation in response to soil inoculum from genetically distant conspecifics.

Seedling survival also displayed maternal identity–specific trends. In *Bactris setulosa* (Figure S6i), survival probabilities increased with genetic distance for seedlings from adults 1, 4, and 7 (Pearson correlations: *r* = 0.39, *p* = 0.002; *r* = 0.37, *p* = 0.005; *r* = 0.55, *p* < 0.001), whereas seedlings from adults 5 and 7 experienced significantly reduced survival under the same conditions (Pearson correlations: *r* = –0.38, *p* = 0.004; *r* = –0.32, *p* = 0.015). Comparable adult-specific survival variation was also noted in *Castilla elastica*, *Castilla tunu*, *Inga marginata*, *Socratea exorrhiza*, and *Vismia sprucei* (Figures S6j, S6k., S6m, S6n and S6o).

**Supplementary Figure S6a: Relationship between total biomass and genetic distance between the maternal source and soil inoculum source in *Bactris setulosa***. Each panel represents seedlings grouped by maternal identity (Adults 1–7). The blue line indicates the fitted linear regression, and the grey shaded area represents the 95% confidence interval. Pearson correlation coefficients (*R*) and associated p-values are shown in each panel.

**Supplementary Figure S6b: Relationship between total biomass and genetic distance between the maternal source and soil inoculum source in *Castilla elastica***. Each panel represents seedlings grouped by maternal identity (Adults 1–6). The blue line indicates the fitted linear regression, and the grey shaded area represents the 95% confidence interval. Pearson correlation coefficients (*R*) and associated p-values are shown in each panel.

**Supplementary Figure S6c: Relationship between total biomass and genetic distance between the maternal source and soil inoculum source in *Castilla tunu***. Each panel represents seedlings grouped by maternal identity (Adults 3, 5-8). The blue line indicates the fitted linear regression, and the grey shaded area represents the 95% confidence interval. Pearson correlation coefficients (*R*) and associated p-values are shown in each panel.

**Supplementary Figure S6d: Relationship between total biomass and genetic distance between the maternal source and soil inoculum source in *Inga edulis***. Each panel represents seedlings grouped by maternal identity (Adults 1–8). The blue line indicates the fitted linear regression, and the grey shaded area represents the 95% confidence interval. Pearson correlation coefficients (*R*) and associated p-values are shown in each panel.

**Supplementary Figure S6e: Relationship between total biomass and genetic distance between the maternal source and soil inoculum source in *Inga marginata***. Each panel represents seedlings grouped by maternal identity (Adults 1–8). The blue line indicates the fitted linear regression, and the grey shaded area represents the 95% confidence interval. Pearson correlation coefficients (*R*) and associated p-values are shown in each panel.

**Supplementary Figure S6f: Relationship between total biomass and genetic distance between the maternal source and soil inoculum source in *Socratea exorrhiza***. Each panel represents seedlings grouped by maternal identity (Adults 1–8). The blue line indicates the fitted linear regression, and the grey shaded area represents the 95% confidence interval. Pearson correlation coefficients (*R*) and associated p-values are shown in each panel.

**Supplementary Figure S6g: Relationship between total biomass and genetic distance between the maternal source and soil inoculum source in *Vismia sprucei***. Each panel represents seedlings grouped by maternal identity (Adults 1–5). The blue line indicates the fitted linear regression, and the grey shaded area represents the 95% confidence interval. Pearson correlation coefficients (*R*) and associated p-values are shown in each panel.

**Supplementary Figure S6h: Relationship between total biomass and genetic distance between the maternal source and soil inoculum source in *Wettinia aequalis***. Each panel represents seedlings grouped by maternal identity (Adults 1–8). The blue line indicates the fitted linear regression, and the grey shaded area represents the 95% confidence interval. Pearson correlation coefficients (*R*) and associated p-values are shown in each panel.

**Supplementary Figure S6i: Relationship between seedling survival and genetic distance between the maternal source and soil inoculum source in *Bactris setulosa***. Each panel represents seedlings grouped by maternal identity (Adults 1–7). The blue line indicates the fitted linear regression, and the grey shaded area represents the 95% confidence interval. Pearson correlation coefficients (*R*) and associated p-values are shown in each panel.

**Supplementary Figure S6j: Relationship between seedling survival and genetic distance between the maternal source and soil inoculum source in *Castilla elastica***. Each panel represents seedlings grouped by maternal identity (Adults 1–6). The blue line indicates the fitted linear regression, and the grey shaded area represents the 95% confidence interval. Pearson correlation coefficients (*R*) and associated p-values are shown in each panel.

**Supplementary Figure S6k: Relationship between seedling survival and genetic distance between the maternal source and soil inoculum source in *Castilla tunu***. Each panel represents seedlings grouped by maternal identity (Adults 3, 5–8). The blue line indicates the fitted linear regression, and the grey shaded area represents the 95% confidence interval. Pearson correlation coefficients (*R*) and associated p-values are shown in each panel.

**Supplementary Figure S6l: Relationship between seedling survival and genetic distance between the maternal source and soil inoculum source in *Inga edulis***. Each panel represents seedlings grouped by maternal identity (Adults 1–8). The blue line indicates the fitted linear regression, and the grey shaded area represents the 95% confidence interval. Pearson correlation coefficients (*R*) and associated p-values are shown in each panel.

**Supplementary Figure S6m: Relationship between seedling survival and genetic distance between the maternal source and soil inoculum source in *Inga marginata***. Each panel represents seedlings grouped by maternal identity (Adults 1–8). The blue line indicates the fitted linear regression, and the grey shaded area represents the 95% confidence interval. Pearson correlation coefficients (*R*) and associated p-values are shown in each panel.

**Supplementary Figure S6n: Relationship between seedling survival and genetic distance between the maternal source and soil inoculum source in *Socratea exorrhiza***. Each panel represents seedlings grouped by maternal identity (Adults 1–8). The blue line indicates the fitted linear regression, and the grey shaded area represents the 95% confidence interval. Pearson correlation coefficients (*R*) and associated p-values are shown in each panel.

**Supplementary Figure S6o: Relationship between seedling survival and genetic distance between the maternal source and soil inoculum source in *Vismia sprucei***. Each panel represents seedlings grouped by maternal identity (Adults 1–5). The blue line indicates the fitted linear regression, and the grey shaded area represents the 95% confidence interval. Pearson correlation coefficients (*R*) and associated p-values are shown in each panel.

**Supplementary Figure S6p: Relationship between seedling survival and genetic distance between the maternal source and soil inoculum source in *Wettinia aequalis***. Each panel represents seedlings grouped by maternal identity (Adults 1–8). The blue line indicates the fitted linear regression, and the grey shaded area represents the 95% confidence interval. Pearson correlation coefficients (*R*) and associated p-values are shown in each panel.

**Supplementary Figure S6q. Relationship between genetic diversity (π) and the strength of intraspecific density dependence (iDD) across species.** Each point represents one species (n = 5). The y-axis shows the species-specific regression slope describing the relationship between genetic distance and either seedling survival or seedling performance, quantified as total biomass, as shown in Figure 4 of the main manuscript). These slopes were used as proxies for the strength and direction of iDD. Positive slope values correspond to stronger negative iDD, while negative slopes indicate positive iDD. Pearson correlation analyses revealed (**a**) a strong, significant positive relationships between genetic diversity (*π*) and iDD strength for plant performance measured from total biomass (*r* = 0.92, *p* = 0.024) and **(b)** non-significant positive relationship between genetic diversity (*π*) and survival-based iDD strength (*r* = 0.57, *p* = 0.32).

References:

1.	Kassambara A. ggpubr: “ggplot2” Based Publication Ready Plots [Internet]. 2025 [cited 2025 Jul 9]. Available from: https://cran.r-project.org/web/packages/ggpubr/index.html
